# Multifocal Tubercular Osteomyelitis with Tubercular Breast Abscess: An Atypical Presentation of Tuberculosis

**DOI:** 10.1155/2015/629141

**Published:** 2015-05-04

**Authors:** Mita Bar, Tuhin Santra, Pradipta Guha, Neha Agrawal, Apu Adhikary, Anirban Das, Chanchal Mahapatra

**Affiliations:** ^1^Department of Medicine, Calcutta National Medical College & Hospital, Kolkata, India; ^2^Department of Medicine, North Bengal Medical College & Hospital, Sushrutanagar, India; ^3^Department of Medicine, Canning Subdivisional Hospital, Canning, India; ^4^Department of Medicine, KIMS Medical College & Hospital, Amalapuram, India

## Abstract

Tuberculosis of spine is common in a developing country like India. However, involvement of spine at multiple levels along with involvement of rib and tubercular breast abscess in an immunocompetent patient without any pulmonary involvement is extremely rare. Here we report a case of 53-year-old immunocompetent lady who presented with quadriparesis and MRI (magnetic resonance imaging) of spine revealed multiple lesions involving cervical, thoracic, lumbar, and sacral region without any involvement of intervertebral disc. On detailed examination she was found to have a lump in right breast. Fine needle aspiration cytology of both paravertebral collection and breast lump revealed presence of acid fast bacilli. She was put on antitubercular drug for one year and she responded well to therapy.

## 1. Introduction

Tuberculosis of spine and rib and tubercular breast abscess are the form of extrapulmonary tuberculosis. Tuberculosis of bone occurs in 10% of patients with extrapulmonary TB (tuberculosis) [1] and spinal TB is the commonest form of skeletal TB [2]. Lower thoracic and lumbar vertebrae are the commonest sites for spinal TB followed by middle thoracic and cervical vertebrae [2]. Usually two or more contiguous vertebrae are involved due to hematogenous spread through one intervertebral artery feeding two adjacent vertebrae [3]. Typically intervertebral disc space along with adjacent vertebral bodies is involved. Cases of spinal TB with intervertebral disc sparing have been reported [4]. However, extensive involvement of all spinal level with extraspinal involvement is extremely rare [5–7]. Also, very few cases of skeletal TB involve ribs [8].

Breast tissue along with skeletal muscles and spleen is relatively resistant to tuberculosis infection [9]. Tuberculosis of breast is very rare even in countries where TB is endemic. Prevalence of breast TB in India varies between 0.64 and 3.59% [10, 11]. Pregnant and lactating women are more susceptible to develop breast TB due to increased vascularity with dilated ducts and it is relatively uncommon in older women [12]. The condition may mimic pyogenic breast abscess or carcinoma of breast both clinically and radiologically [13]. It is mainly of two types, primary and secondary. Primary form is very rare. Secondary form is seen more frequently and main routes of spread are hematogenous, retrograde spread from axillary lymph nodes and direct extension from lung, pleura, mediastinum, costa, and sternum [14, 15].

Presence of multifocal tubercular osteomyelitis and breast TB may cause diagnostic difficulty as the condition mimics malignancy and radiological investigation often fails to differentiate between malignancy and infection. Diagnosis needs to be confirmed by histological examination.

## 2. Case Report

A 53-year-old nondiabetic and nonhypertensive female patient who was having an insidious onset and gradually progressive weakness of all four limbs for past two months along with decreased sensation in both lower limbs and tingling sensation in all four limbs now presented to us with development of retention of urine for the last four days. Two months back she had a preceding history of low grade fever for about one week which subsided spontaneously but she developed a persistent dull aching neck pain along with a gradually progressive quadriparesis. She had no history of anorexia, weight loss, cough with expectoration, hemoptysis, or any lump elsewhere in body. Examination of general survey was unremarkable except for the presence of mild pallor. Neurological examination revealed grade 3 power in both lower limbs and grade 4 power in both upper limbs, spasticity of all four limbs with exaggerated deep tendon reflexes in all 4 limbs, and bilateral extensor type of plantar response and hypoesthesia of both lower limbs but there was no definite sensory level. There was mild hepatosplenomegaly.

**Figure 1:**
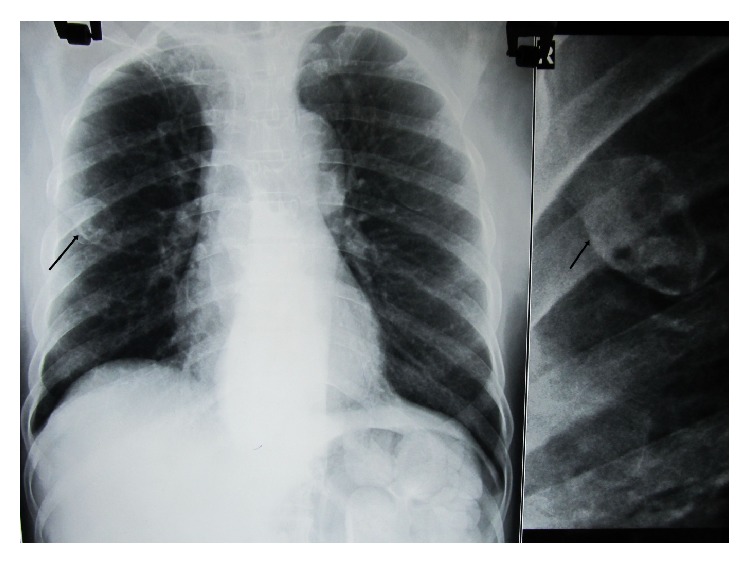
Arrowhead showing an expansile lesion involving right 4th rib.

**Figure 2:**
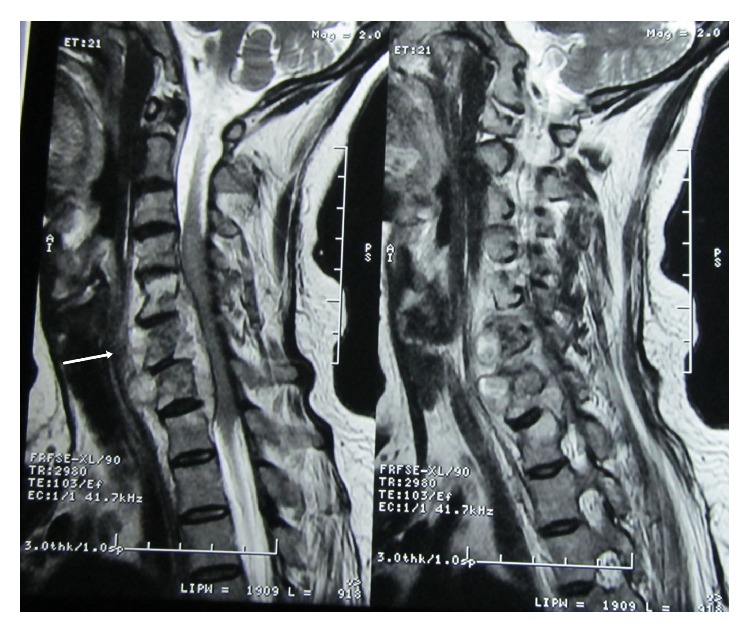
MRI of cervicodorsal spine showing altered marrow signal intensity with erosion of C5 to D1 vertebrae and compression of spinal cord.

**Figure 3:**
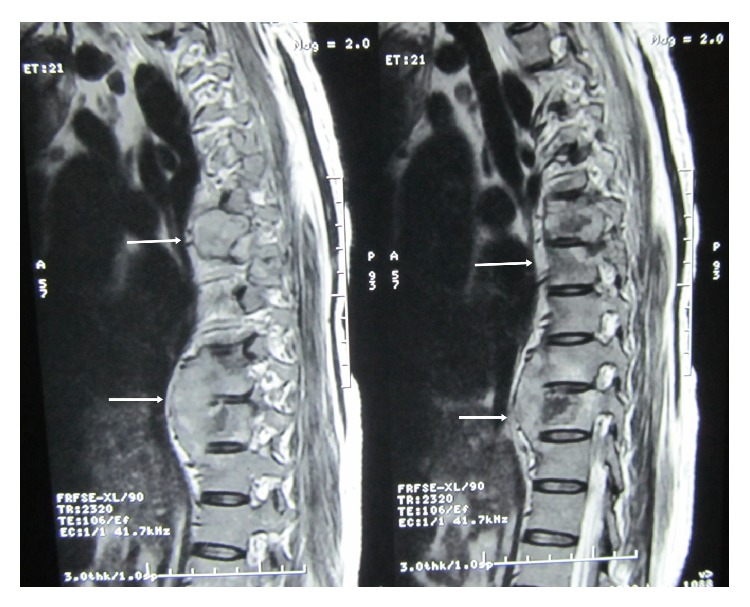
MRI of dorsal spine showing altered marrow signal intensity with abnormal soft tissue lesion involving D6, D7, D9, and D10 vertebrae and intraspinal extension with thecal sac compression at D6 vertebra.

**Figure 4:**
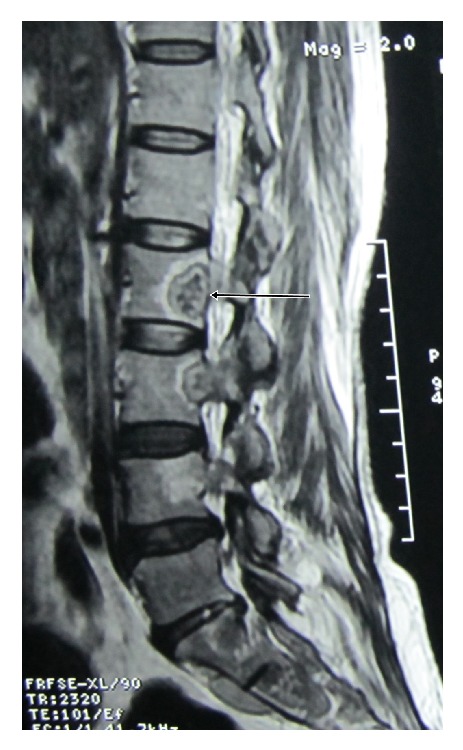
Arrowhead showing altered marrow signal intensity with abnormal soft tissue lesion at L2 vertebra.

Her routine blood investigation revealed a hemoglobin level of 8.1 g/dL, total leucocyte count was 8,000/mm^3^, and ESR (erythrocyte sedimentation rate) was 42 mm in 1st hour. Blood sugar, urea, creatinine, and liver function test were within normal limit. She was found to be HIV (human immunodeficiency virus) negative. Her chest X-ray showed an expansile lesion involving right 4th rib (Figure 1). MRI of spine revealed altered marrow signal intensity with erosion of C5 to D1 vertebrae. There was intraspinal extension of the disease process with elevation of posterior longitudinal ligament between C5 and D1 levels causing compression of thecal sac and spinal cord. The intervertebral discs were of normal height and signal intensity (Figure 2). MRI of dorsal and lumbar spine showed altered marrow signal intensity with abnormal soft tissue lesion involving D6, D7, D9, D10, and L2 to L5 vertebrae and sacrum. Pedicles at D10 and L5 level are also involved. Intraspinal extension with thecal sac compression is seen at the level of D6 vertebrae and in sacrum. Large pre- and paravertebral soft tissue element are seen at dorsal level and presacral space (Figures 3 and 4).

**Figure 5:**
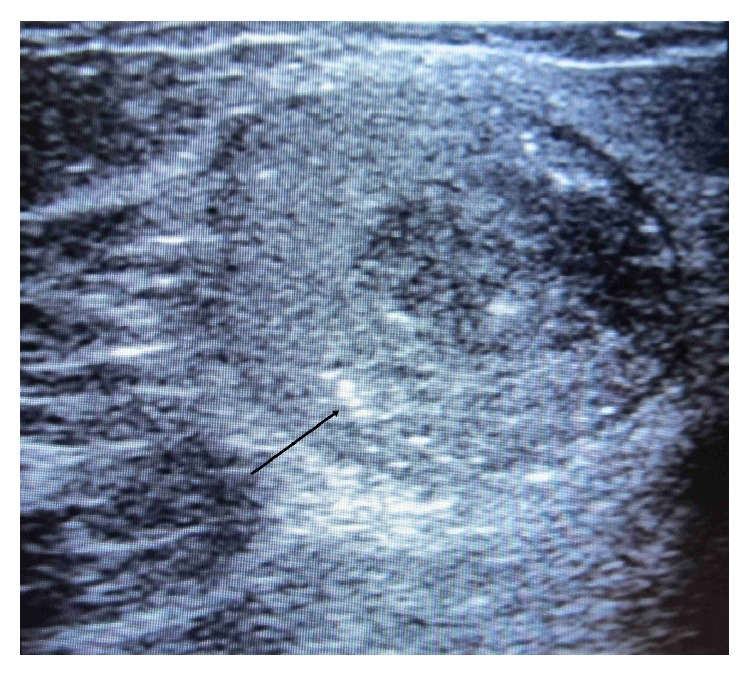
USG of right breast showing hypoechoic, heterogeneous mass lesion with echogenic foci (arrowhead).

**Figure 6:**
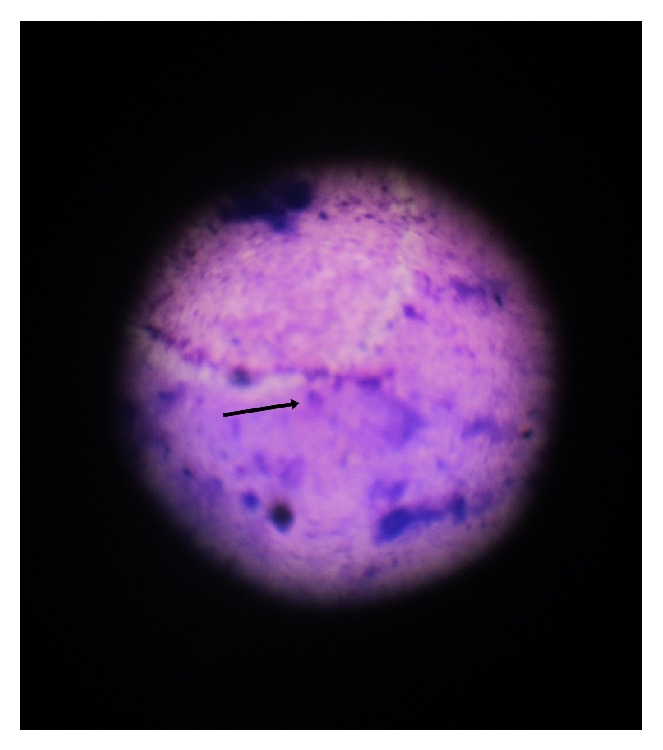
FNAC from breast lesion showing degenerated inflammatory cells in a necrotic background.

**Figure 7:**
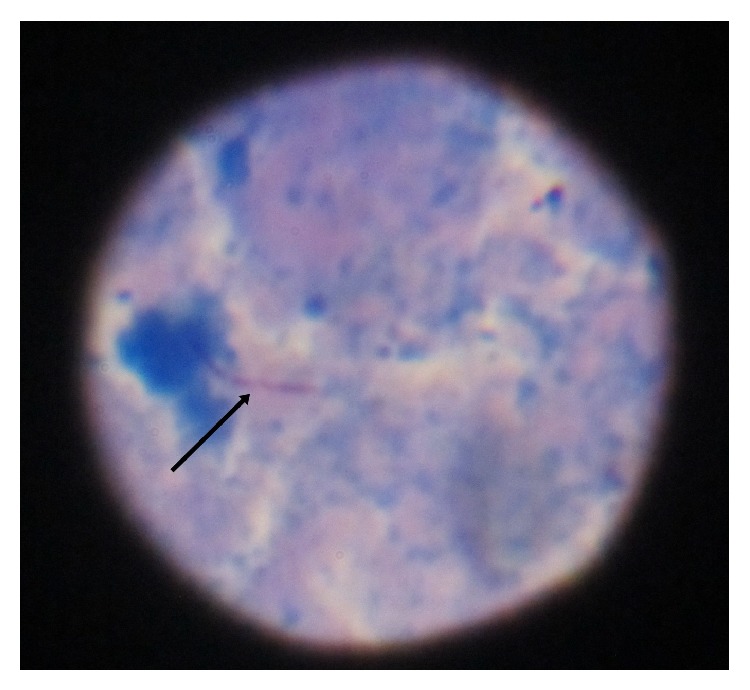
ZN stain of FNAC material from breast tissue with arrowhead showing acid fast bacilli.

At this time we had a differential diagnosis of some metastatic disease process or tuberculosis. We examined the patient once again and this time we found a small breast lump involving upper outer quadrant of right breast which was painless, mobile, and firm in consistency which was unnoticed by the patient. This raised the possibility of carcinoma of breast with metastasis. We went for USG (ultrasonography) of breast which showed a hypoechoic, heterogeneous mass lesion involving right breast measuring 3.5 × 2.3 cm (Figure 5) with echogenic foci within it. Few enlarged axillary lymph nodes are noted on right side, the largest of which was 2 × 0.8 cm USG of left breast was normal. Next, we went for CT (computed tomography) guided FNAC (fine needle aspiration cytology) from paravertebral collection and USG guided FNAC from right breast lesion (Figure 6) both of which showed degenerated inflammatory cells in a necrotic background without any presence of malignant cell or granuloma. However, ZN (Ziehl-Neelsen) stain of both lesions showed presence of acid fast bacilli (Figure 7) which was suggestive of tubercular lesion.

Patient was put on antitubercular drug for 1 year (initial 2 months, rifampicin, isoniazid, pyrazinamide, and ethambutol, followed by rifampicin and isoniazid for the next 10 months). After treatment her quadriparesis improved completely with resolution of breast lump and rib lesion.

## 3. Discussion

Immunocompromised patients are at higher risk of developing extrapulmonary tuberculosis. Prevalence is higher in HIV infected patients, patients on immunosuppressive therapy, and hemodialysis patients [16]. Multifocal skeletal TB is less common in immunocompetent patients even in countries where TB is endemic. The insidious nature of disease process may lead to delayed diagnosis or misdiagnosis. Usually two distinct patterns of spinal TB have been identified based on radiological imaging: the first pattern which is more common involves intervertebral disc and adjacent vertebral bodies; the second pattern involves body or neural arch of one or more vertebrae with sparing of intervertebral disc and it is seldom seen [4]. Tubercular osteomyelitis of rib is also rare. The ribs are involved in only 0.1% of all tuberculosis infections [8]. Tuberculosis of breast which commonly affects young women in their reproductive age group (21 to 30 years) is uncommon in elderly women [12, 17].

Presence of breast lump with multiple spinal lesions mimics a malignant process. Our case shows an extremely rare form of tuberculosis where the disease process affected spine at multiple levels with intervertebral disc sparing along with involvement of breast tissue and rib and the condition initially gave rise to a possibility of malignancy of breast with metastasis. FNAC was helpful to diagnose the condition and culture was not done as presence of AFB in ZN stain in this type of clinical scenario hints at a diagnosis of tuberculosis infection. However, due to the increasing evidence of drug resistant* Mycobacterium* infection, culture and sensitivity test may be necessary for all tuberculosis cases.

Breast tuberculosis usually occurs from hematogenous spread or retrograde spread from axillary lymph nodes or direct extension from underlying structure like lung, pleura, and costa [14, 15]. In our case multiple locations of pathological process give rise to a possibility of hematogenous spread of tuberculosis infection. However, breast involvement might be due to retrograde axillary spread as this type of spread usually favours upper outer quadrant of breast [17] as what has occurred in our case or it might be due to spread from underlying infected rib both of which are a possible mechanism.

So far there is only one reported case of dual location of breast and spine TB [7] and that was reported from outside India. To the best of our knowledge, this is the first ever case of multifocal spinal and breast tuberculosis along with rib involvement, without any pulmonary involvement in an immunocompetent patient from a country like India where TB is endemic.

## 4. Conclusion

A differential diagnosis of tuberculosis should be considered in mind whenever a patient presents with lesion at multiple spinal levels with intervertebral disc sparing. Presence of a breast lump should be evaluated carefully. Clinical and radiological findings may not be helpful for proper diagnosis but may be helpful in assessing extent of disease involvement. Histological examination is necessary for proper diagnosis.

## Abbreviations

TB: Tuberculosis
ESR: Erythrocyte sedimentation rate
HIV: Human immunodeficiency virus
MRI: Magnetic resonance imaging
USG: Ultrasonography
cm: Centimeter
mm: Millimeter
CT: Computed tomography
FNAC: Fine needle aspiration cytology
ZN: Ziehl-Neelsen.

### Vertebral Level

C: Cervical
D: Dorsal
L: Lumbar.
